# Heterodimeric Plasmonic Nanogaps for Biosensing

**DOI:** 10.3390/mi9120664

**Published:** 2018-12-16

**Authors:** Sharmistha Chatterjee, Loredana Ricciardi, Julia I. Deitz, Robert E. A. Williams, David W. McComb, Giuseppe Strangi

**Affiliations:** 1 Department of Physics, Case Western Reserve University, 10600 Euclid Avenue, Cleveland, OH 44106, USA; chatterjee.bwd@gmail.com; 2 CNR-NANOTEC Istituto di Nanotecnologia and Department of Physics, University of Calabria, 87036 Rende, Italy; loredana.ricciardi@unical.it; 3 Fondazione con Il Cuore, via Roma 170, 88811 Ciro’ Marina, Italy; 4 Center for Electron Microscopy and Analysis, The Ohio State University, Columbus, OH 43212, USA; deitz.13@osu.edu (J.I.D.); williams.2156@osu.edu (R.E.A.W.); mccomb.29@osu.edu (D.W.M.); 5 Department of Material Science and Engineering, The Ohio State University, Columbus, OH 43210, USA

**Keywords:** plasmonic nanostructures, EELS, TEM, FEM, nanogaps, hot-spot, single molecule sensing

## Abstract

We report the study of heterodimeric plasmonic nanogaps created between gold nanostar (AuNS) tips and gold nanospheres. The selective binding is realized by properly functionalizing the two nanostructures; in particular, the hot electrons injected at the nanostar tips trigger a regio-specific chemical link with the functionalized nanospheres. AuNSs were synthesized in a simple, one-step, surfactant-free, high-yield wet-chemistry method. The high aspect ratio of the sharp nanostar tip collects and concentrates intense electromagnetic fields in ultrasmall surfaces with small curvature radius. The extremities of these surface tips become plasmonic hot spots, allowing significant intensity enhancement of local fields and hot-electron injection. Electron energy-loss spectroscopy (EELS) was performed to spatially map local plasmonic modes of the nanostar. The presence of different kinds of modes at different position of these nanostars makes them one of the most efficient, unique, and smart plasmonic antennas. These modes are harnessed to mediate the formation of heterodimers (nanostar-nanosphere) through hot-electron-induced chemical modification of the tip. For an AuNS-nanosphere heterodimeric gap, the intensity enhancement factor in the hot-spot region was determined to be 10^6^, which is an order of magnitude greater than the single nanostar tip. The intense local electric field within the nanogap results in ultra-high sensitivity for the presence of bioanalytes captured in that region. In case of a single BSA molecule (66.5 KDa), the sensitivity was evaluated to be about 1940 nm/RIU for a single AuNS, but was 5800 nm/RIU for the AuNS-nanosphere heterodimer. This indicates that this heterodimeric nanostructure can be used as an ultrasensitive plasmonic biosensor to detect single protein molecules or nucleic acid fragments of lower molecular weight with high specificity.

## 1. Introduction

Real-time and label-free detection of protein molecules at ultralow concentration in their natural state is considered a longstanding need in biomedical research, as it could allow for the identification of the onset of cancers before they become clinically relevant, to monitor disease progression and evaluate therapeutic success, thereby increasing survival rates and quality of life [1]. However, the detection of single protein molecules is extremely challenging because of their acutely small size (<3 nm) [2]. One way to detect these proteins is to use the unusual electromagnetic responses of electron clouds of noble metal nanoparticles (NPs), which leads to the well-known localized surface plasmon resonance (LSPR) effect. This makes NPs suitable for a large variety of applications [3], such as surface enhanced Raman spectroscopy (SERS) [4,5], sensing, [6,7] subwavelength imaging [8], photocatalysis [9], photovoltaics [10,11], phototherapies [12], quantum technologies, and miniaturized photonic circuits, and for on-chip integration of photonic and electronic systems [13,14]. Light confinement at LSPR can be tuned by controlling the nanoparticle size, shape, material, and dielectric matrix in which the plasmon nanostructure is located [15,16]. Core shell nanoparticles are better than rigid spherical nanoparticles for their higher tunability and electric field enhancement capability [17]. However, it has been observed that NPs with sharp edges, such as nanotriangles [18], nanocubes [19,20], nanorods [21,22], nanostars [23,24,25,26], or octahedral nanoparticles [27,28], have a higher capability of confining light in ultra-small regions compared to the non-spiky spherically symmetric NPs. The enhanced electromagnetic energy concentration at the hot spots of spiky NPs is due to the plasmonic resonance effect, which is more effective than the non-spiky nanoparticles because of their lightning rod effect [29,30,31,32] and the small radius of curvature of the edges, which results in the enhancement of the local electric field, distributed over the hot spot area of the nanostructure. Because of the wide application of spiked gold NPs, the effective and controlled synthesis parameters that can tune LSPRs along with the ability to manipulate hot spot landscapes has become an important research field.

Recently, significant efforts have been devoted to achieve novel nanomaterials that allow for the manipulation of the interaction of light at the nanoscale. Gold is the most widely used noble metal for biomedical applications due to its well-known low toxicity, biocompatibility, and tunability. The popular wet-chemistry method represents a largely used bottom-up approach for NP synthesis among many other possible routes. Furthermore, elongated nanoantennas synthesized via a surfactant-free route will be beneficial not only for in vitro sensing but also for in vivo testing. Until recently, very few research groups have synthesized spiked nanoantennas via a surfactant-free wet-chemistry route. Among all the possible spiked antennas such as the nanoellipsoid and the nanorod, which can concentrate light like nanostars [33,34,35,36,37,38], gold nanostars (AuNSs) with a random distribution of spikes over their core have the advantage of having hot spots excited by any kind of polarization of the incident light, being polarization-insensitive. The higher probability of generation of a few hot spots irrespective of incident light polarization as well as the creation of huge intensity enhancement in the hot spots make these highly tunable AuNSs very promising in the field of real-time biosensing at a point of care with respect to gold nanorods and nanoellipsoides. Different research groups have synthesized AuNSs in many different ways, including green synthesis routes [39,40,41,42,43,44,45], but there is still a need for a low-cost, simple, surfactant-free wet chemistry method for synthesizing these highly efficient spiked nanoplasmonic antennas with high yield. Here, we report a simple, one-step surfactant-free wet chemistry method for synthesizing Au nanostructures with high stability and yield. This improves on our previously reported AuNS synthesis method [46], where the stability was poor and the control of the nanostar synthesis parameters was not possible. Synthesis as well as optical and electron spectroscopy characterization of these highly stable nanoparticles (stability > 5 months in aqueous solution) indicate that these nanoparticles have remarkable plasmonic features. The optical characterization provides information regarding the collective behavior of the nanostars present in the aqueous solution, whereas electron energy-loss spectroscopy (EELS) investigations performed in the scanning transmission electron microscope (STEM) allows for high-resolution spatial determination of the local plasmonic response such as the tip, the core of the nanostar, and different portions of the spike. EELS reveals the presence of different kinds of modes in these AuNSs, which makes them unique and more efficient than the existing nanoantennas. Based on this information, heterodimeric nanogaps can be created at any region of the nanostars by merely choosing the specific mode of that region. The observed breathing modes that originate from the confinement of surface plasmons by the geometrical boundaries of a nanoparticle are very efficient for any kind of near field coupling because of their higher optical mode density and are thus important for the desired sensing experiments [47]. Previously, several groups have reported EELS measurements on various nanoantennas including nanostars, but none of them reported about the breathing modes of the nanostars [18,48,49,50,51,52,53,54,55,56,57,58,59,60]. The EELS studies and the relevant numerical investigations have provided key information for designing controlled heterodimeric nanogaps by using a regio-selective surface chemistry method mediated by hot electrons, which was introduced by Cortés, E. et al. [61] Theoretical investigations have also been carried out, confirming both the collective as well the individual AuNS behavior in aqueous solution. The maximum intensity enhancement and the sensitivity for the single AuNS and for the hybrid AuNS-nanosphere system have also been calculated using the finite element method (FEM).

## 2. Experimental Section

**Materials:** Gold (III) chloride trihydrate (HAuCl_4_, 3H_2_O), silver nitrate (AgNO_3_), ascorbic acid (AA), hydrochloric acid (HCl) (35–37%), and polyvinylpyrrolidone (PVP) were purchased from Sigma Aldrich (Milan, Italy)and used as received without further purification. The water used throughout this synthesis process was reagent-grade, produced using a Milli-Q SP ultrapure-water purification system.

**Synthesis of the Stabilized Gold Nanostars:** Here gold nanostars were synthesized in a simple, one-step (without seed), surfactant free wet-chemistry method. First, 10 mL of 0.25 mM chloroauric acid (HAuCl_4_) solution [in presence of 10 μL of 1 M HCl solution] was taken in a 20 mL glass vial. The solution was stirred at room temperature under moderate stirring (700 rpm). Then, 100 μL of AgNO_3_ solution of 1 mM concentration and 50 μL of AA solution of 100 mM concentration were added simultaneously with the above chloroauric acid solution at room temperature under moderate stirring (700 rpm). The color of the solution rapidly became blue within 30 s and after 2 min from the addition of the AA and AgNO_3_, and 5 mL of polyvinylpyrrolidone (PVP) solution at a 2 mM concentration was added and the solution was stirred for 8 min. After 10 min as a whole, the reaction was completed and the solution was kept for another 3 h at room temperature at rest. After that, one centrifugal wash had been done at 4000 rcf for 20 min in a 15 mL tube to wash out the extra PVP. After centrifugation, liquid containing extra PVP and the other chemicals was collected as much as possible, and the precipitate was redispersed in DI water. In this way, we got our stabilized AuNSs dispersed in DI water and kept it at room temperature for future use. Here in this synthesis process, the length and number of spikes of nanostars could be easily adjusted by varying pH, stirring speed, and concentration ratios of the ingredients. Here we have maintained a constant stirring speed throughout the synthesis process. The synthesized AuNSs were well characterized and these details are given below.

**Characterization:** UV-Vis-NIR spectroscopy (Agilent, Cary, NC, USA) and transmission electron microscopy TEM (FEI, Hillsboro, OR, USA) were applied to characterize the synthesized nanoparticles.

**UV-Vis-NIR Spectra:** A Perkin Elmer Lambda 900 spectrophotometer (Perkin Elmer, Shelton, CT, USA) was used to obtain the UV-Vis-NIR spectra of the synthesized gold nanostars solution, which describes the extinction property of this nanostar solution in a wavelength range of 400–1300 nm.

**High-Resolution STEM, EDS, and EELS Measurements:** Scanning transmission electron microscopy (STEM, FEI Thermo Fisher Scientifics, Hillsboro, OR, USA) (monochromated, aberration-corrected FEI Titan^3^ G2 STEM) was used to probe the size and shape of the synthesized gold nanoparticles. Low-loss EELS was employed to elucidate the electronic structure of the synthesized gold nanoparticles, while X-ray energy dispersive spectroscopy (XEDS) was used to determine the weight percentage of the gold present in the solution. Samples were prepared by depositing aqueous suspension containing the synthesized gold nanoparticles on a standard holey carbon film supported by a TEM grid which was left drying in air one day before the STEM measurements. All work was performed at 60 kV with a high collection angle (approximately 25 mrad) to minimize Cherenkov radiation in the EELS signal [62]. The convergence angle was 13.2 mrad, and the probe size was approximately 1.3 angstrom. The EELS energy resolution was approximately 150 meV (full width at half-maximum of the zero-loss peak). Spectrum imaging was used to spatially resolve the EELS signal along and across each nano-object, and all EELS data were processed using the Gatan Digital Micrograph software package. During the EEL spectra acquisition, there was no evidence of irradiation damage in the samples. The zero-loss peak for each spectrum was removed using the standard reflected tail method, which reflects the tail on the energy-gain side of the spectrum onto the energy-loss side, typically with a predefined scaling factor, and subtracts it [63,64,65,66].

**Numerical Simulation:** FEM simulations are used here to study the interaction of a plasmonic biosensor of a single AuNS and a hybrid AuNS-nanosphere with photons. All the data regarding nanostars spikes and their cores are taken from the TEM studies. The nanostructure’s surface is discretized with tetrahedral mesh elements with a typical maximum and minimum side lengths of 24.5 and 1.05 nm, respectively. During simulations, the electric field was mapped in the entire space. Photon interaction with single AuNS and AuNS-nanosphere heterodimer with a different nanosphere size and a gap distance were studied. Different cross sections (Scattering, Absorption and Extinction) of those nanoantennas were also examined. During sensitivity calculation, we considered first air (ε = 1.0) and then water (ε = 1.7689) as the homogeneous surrounding media. In all simulations, the wavelength-dependent permittivity of the gold was taken from [67]. We also calculated the shift in LSPR of the single AuNS and the AuNS-nanosphere heterodimer when a BSA molecule (of 0.11 attogram mass) is coupled in the hot spot region of those nanoantennas immersed in water medium. During simulation, a BSA protein molecule was taken as a dielectric particle of cylindrical shape with a permittivity of 2.25, with a characteristic height of 3.4 nm and a 6.8 nm short cylinder diameter [68].

## 3. Results and Discussion

To obtain nanostars in aqueous suspension with high yield and stability, there are parameters that need to be precisely controlled during synthesis. These parameters are summarized in this section (see Methods and Supporting Information for more details). One of these parameters is the relative amount of reducing agent to the gold precursor. Here, ascorbic acid (AA, C_6_H_8_O_6_) was used as the reducing agent, and chloroauric acid (HAuCl_4_) was used as the gold precursor. The ratio of AA to chloroauric acid was maintained to be 1.5–2.0 to reduce all HAuCl_4_ molecules present in the solution completely. Secondly, the presence of Ag^+^ (AgNO_3_) was necessary in this synthesis process for nanostar formation. During synthesis, the main function of Ag^+^ was to expedite the anisotropic growth of Au branches on certain crystallographic facets. The synthesis procedure will only yield polydispersed nanorods and nanospheres in the absence of Ag^+^ (AgNO_3_). Increasing the amount of AgNO_3_ during the synthesis will increase the length and the number of spikes of the AuNS (see Figure S1). The third factor was the presence of HCl. A small amount of HCl helped in this case to slightly decrease the pH of the solution and a red-shifted plasmon band [33]. The fourth factor was the simultaneous injection of AA and AgNO_3_, in the HAuCl_4_ solution (in the presence of HCl), because this will significantly influence the polydispersity, yield, and quality of the nanostars. The fifth factor was the stirring speed. A study to probe the best stirring speed found that 700 rpm was the best for a successful synthesis as the measured extinction cross section for the AuNS solution is highest for that speed for any specific bar (see Figure S2). The sixth factor was the injection time of polyvinylpyrrolidone (PVP). The best injection time of PVP for a successful synthesis was after 2 min from the time of simultaneous injection of AA and AgNO_3_ (see Figure S3), as by that time the reduction process had quite enough time to be completed and was not affected by the PVP addition by any means. Here, PVP concentration was taken lower so that the reduction kinetics was not affected. PVP was added for creating a hindrance to the nucleation process among the nanostars only for a highly stable AuNS aqueous suspension. The nanostars were found to be stable in aqueous solution for more than five months. During AuNS synthesis and the study of different synthesis parameters, the effect of slight variations in temperature was neglected, as the temperature was never above 60 °C, so any observable effect on the synthesis end product due to temperature variation was not expected [51].

**Figure 1:**
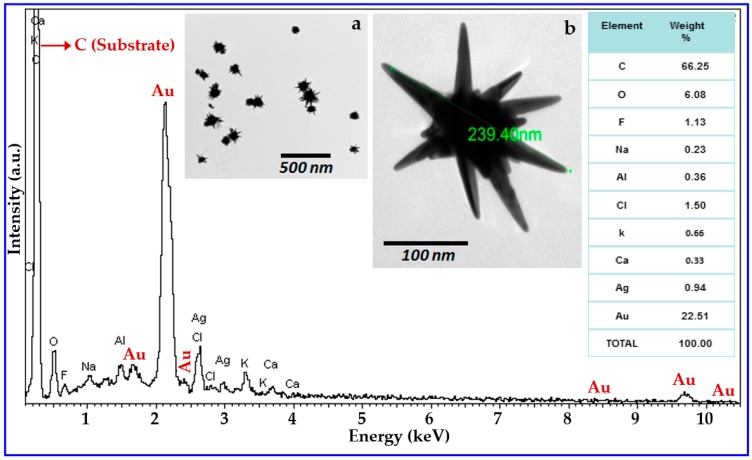
X-ray energy dispersive spectroscopy (XEDS) of the synthesized AuNS solution dispersed on a carbon tape. The inset table shows the weight percentages (%) of the elements present in the sample. Inset Figure ‘a’ shows a typical lower magnification TEM image of a collection of Au nanoparticles, showing that the majority of nanostructures have some spiked areas, confirming the relatively high yield of the synthesis method. Inset Figure ‘b’ is a typical higher magnification transmission electron microscopy (TEM) image of the synthesized nanostructure.

Figure 1 shows the X-ray energy dispersive spectroscopy (XEDS) of the synthesized AuNS solution. In the spectrum, the most intense peak corresponds to carbon due to the carbon tape substrate used. The peak corresponding to Au is the second most intense peak in the spectrum, and the weight percentage of the gold present in our synthesized gold nanoparticle solution is 22.51%. Figure 1a shows a typical lower magnification TEM image where almost all nanoparticles were found to have at least one spike in their surface, confirming the relatively high yield of the synthesis method. Figure 1b shows a randomly selected higher magnification TEM image of a gold nanoparticle.

**Figure 2:**
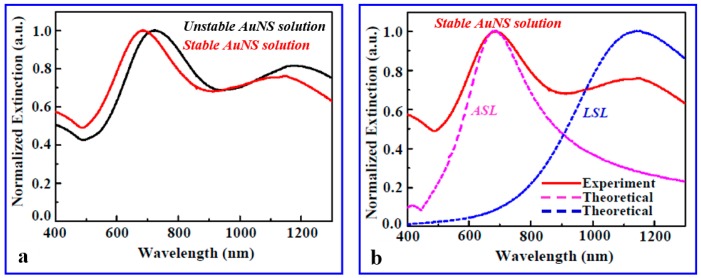
(**a**) The UV-Vis-NIR spectra of the synthesized AuNS solution in both stable and unstable condition. (**b**) Normalized experimental extinction cross section of stabilized AuNS solution and the relevant theoretical investigation of the extinction property of AuNSs with two different spike lengths. The largest spike length (LSL) and average spike length (ASL) were calculated based on the collected TEM information.

Figure 2a shows two extinction spectra for both the stabilized AuNS solution and the AuNS solution that is not stabilized. Figure 2b shows the difference between the normalized experimental extinction characteristics of the stabilized AuNS solution and the corresponding theoretical investigations. Here, two modes are distinctive in the experimental extinction spectrum. As per the data collected from the TEM images of several AuNSs, the average spike length (ASL) of the nanostars was about 63 nm, taking into account all possible spike lengths from the smallest spike length (SSL) of 33 nm to the largest spike length (LSL) of 90 nm, and the average diameter of the core was almost 60 nm. Depending on the length of the spike, the tip radius varied from 5 to 1 nm. FEM simulations were performed for two AuNSs with ASL and LSL spike lengths and with the same core diameter of 60 nm to determine their extinction characteristics. The purple and blue curves in Figure 2b represent the normalized extinction spectra of the theoretical AuNS with ASL and LSL spike lengths, respectively. By considering the convolution of both theoretical curves, we can conclude that the resultant theoretical extinction characteristics match well with the experimental curve profile and the LSP resonances.

**Figure 3:**
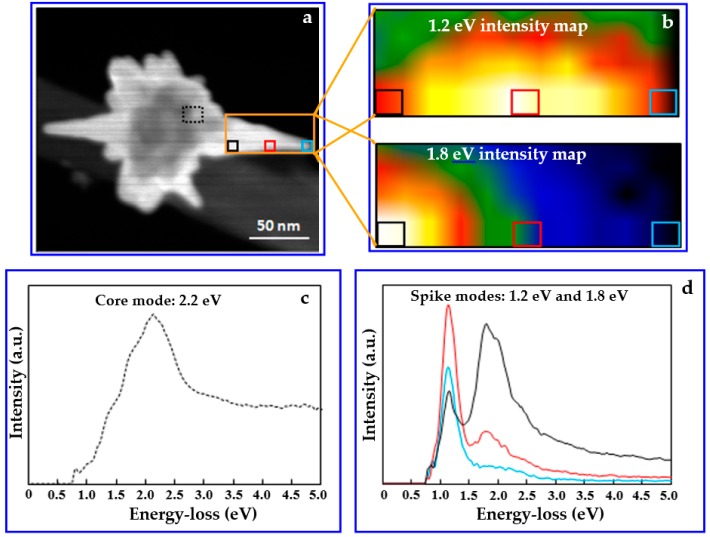
Electron energy loss spectroscopy (EELS) characterization of the AuNS: (**a**) AuNS image with relative areas of investigation (colored boxes); (**b**) intensity maps of major plasmonic modes at 1.2 and 1.8 eV located at the spike of the AuNS; (**c**) EELS spectra of core of the AuNS; (**d**) EELS spectra of different regions of the spike of the AuNS.

Although experimental and theoretical studies of the extinction properties of the AuNS solution provided relevant information about the ensemble behavior of AuNSs in aqueous solution, to move towards a controlled construction of hybrid plasmonic nanogaps for sensing, local information regarding the plasmonic field distribution and resonance frequencies is necessary. To use the regio-specific surface chemistry method based on hot electron injection introduced by Cortés et al. [56] for the creation of heterodimeric nanogaps between AuNSs and Au nanospheres, STEM-EELS investigations of the nanostructure were performed to gain precise information about single AuNS plasmonic modes. Figure 3 illustrates the EELS analysis of the synthesized AuNS.

Figure 3a shows a STEM-HAADF image of the AuNS that was used for EELS analysis. The boxes on the image indicate the regions from which the EELS spectrum images were acquired. Shown in Figure 3c,d are the spectrum images in the center of the nanostar (the black dotted box in Figure 3a) and the spike of the nanostar (colored boxes within the orange box area in Figure 3a).

**Figure 4:**
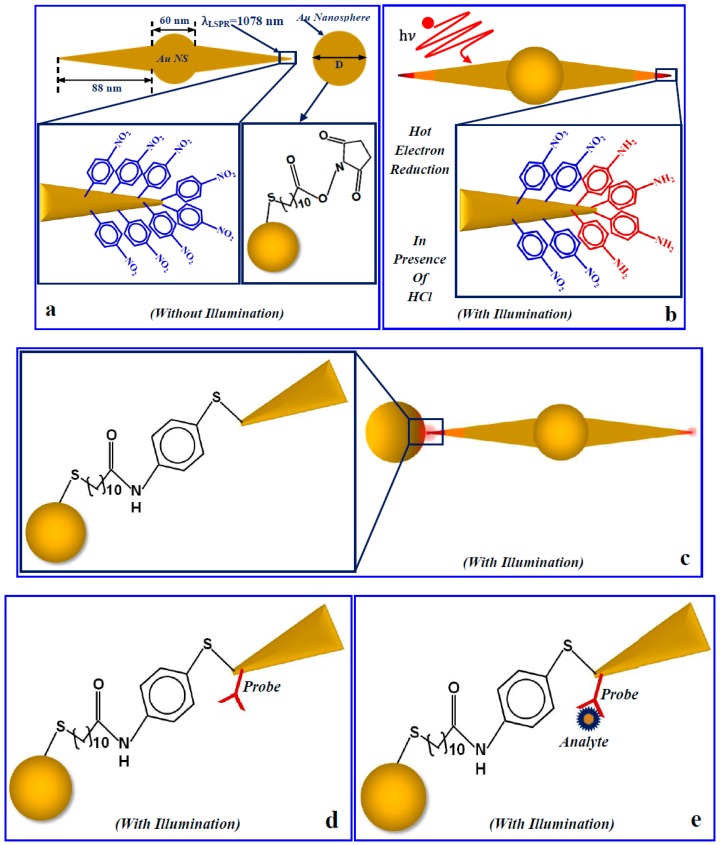
Design of a hybrid AuNS-nanosphere plasmonic antenna via the local surface chemistry modification method. (**a**) AuNS surface modified by 4-NTP and Au nanosphere coated with 11-mercaptoundecanoic acid (MUA) are shown. (**b**) Hot electron injection after light illumination on a 4-NTP-coated AuNS antenna at LSPR wavelength (1078 nm) in the presence of HCl; (**c**) The formation of a hybrid nanoantenna where the Au nanosphere is coupled to the AuNS tip. This happened when the activated and purified Au nanospheres left in contact with the hot-electron-converted AuNS antennas. (**d**) The hybrid plasmonic antenna converted as a sensor by only attaching a suitable probe to this system. (**e**) The plasmonic heterodimer sensor in the presence of a bioanalytes.

In the low energy-loss region, the spectrum exhibits one major peak at 2.2 eV (~564 nm). Numerical investigation of the Au nanosphere with a 60 nm diameter shows LSPR at 550 nm (see Figure S4), suggesting that the 2.2 eV is associated with the LSPR at the core of AuNS. The spectra from the spike (Figure 3d) exhibit two peaks at 1.2 eV (~1033 nm) and 1.8 eV (~689 nm). The relative intensity of the two peaks varies spatially along the spike as shown in the intensity maps of 1.2 eV mode and 1.8 eV mode in Figure 3b. The peak at 1.2 eV shows a maximum intensity at a position approximately halfway along the length of the spike, but the presence of the 1.8 eV mode can also be seen at that location (see Figure S5). At the pinnacle of the 88 nm spike (the region within the cyan box), the major plasmonic resonance is at 1.2 eV. On the other hand, the 1.8 eV mode exhibits maximum intensity at a location closer to the core of the nanostar. FEM investigation on the extinction properties for the AuNSs with an 88 nm spike length and a 60 nm core shows that the LSPR occurs at 1060 nm (see Figure S6). Both the numerical result and the EELS investigation indicate that the mode that is predominating in an AuNS tip region is 1.2 eV, which is crucial information to design regio-specific interactions that allow selected nanostructures to be bound at the tip of the AuNS via the non-localized surface chemistry method. Figure 4 shows how a regio-specific interaction—driven by hot-electron injection—can promote the formation of a heterodimeric (nanostar-nanosphere) nanostructure for plasmonic biosensing via the method introduced by Cortés et al. [61].

The plasmonic biosensor detection mechanism is based on the measurement of the LSPR shift of a nanoantenna when a protein molecule enters the region of space of its plasmon field. According to the sensing principle, when a protein molecule is nearby a plasmonic antenna, it feels an intense attraction toward the hot-spot region because of the force induced by the strong light confinement [68]. Therefore, when a small protein molecule is adsorbed onto the surface of the nanoparticle; the evanescent field starts to polarize the molecule. As the local field at the hot spot of the nanoantenna has to do some work to polarize the molecule the overall energy of the mode reduces accordingly. Experimentally, this situation can be visualized as a shift of the previously observed plasmonic mode of the nanoantenna, which is actually a narrow dip in the transmission spectrum or a sharp peak in the elastic spectrum [68,69,70]. In this case, the local field of the nanogap behaved similarly in the presence of the bio-nanoparticle. The energy of the hybrid mode of the heterodimeric nanogap reduces in presence of a foreign molecule. This reduction in the energy of the hybrid mode was realized from its shift in the transmission spectrum and thus can be used for molecular detection. Here, because of the high electric field enhancement at the binding site (nanogap) and the optimum distribution of the field, the gap mode shift for a single (or a few) molecule of lower molecular weight (such as the BSA protein molecule with 66.5 kDa) is detectable (numerical investigation supports this claim). This detection could be even more specific by attaching a probe molecule at the gap.

For large protein molecules (>1 MDa), energy loss to polarize the molecule is higher, so a larger LSPR shift that is easy enough to detect occurs; however, for the case of a protein molecule of ultrasmall molecular weight and thus smaller polarizability, the loss of energy of the LSPR shift becomes difficult to detect. Recently, single protein molecule detection has been reported using a plasmonic biosensor based on nanorods [69]. The reported S/N was very low, as the FWHM line width of the LSPR spectrum was larger (~50 nm) than the average wavelength shift (~0.1 nm) produced due to the adsorption of a molecule on the surface of the nanoplasmonic particle [69]. Therefore, real-time (label-free) detection of protein molecules of lower molecular weight (e.g., the Thyroglobulin molecule of a 660 kDa molecular weight and a BSA molecule of a 66.5 kDa molecular weight) in ultralow concentration appears to be forbidden with current technologies.

**Figure 5:**
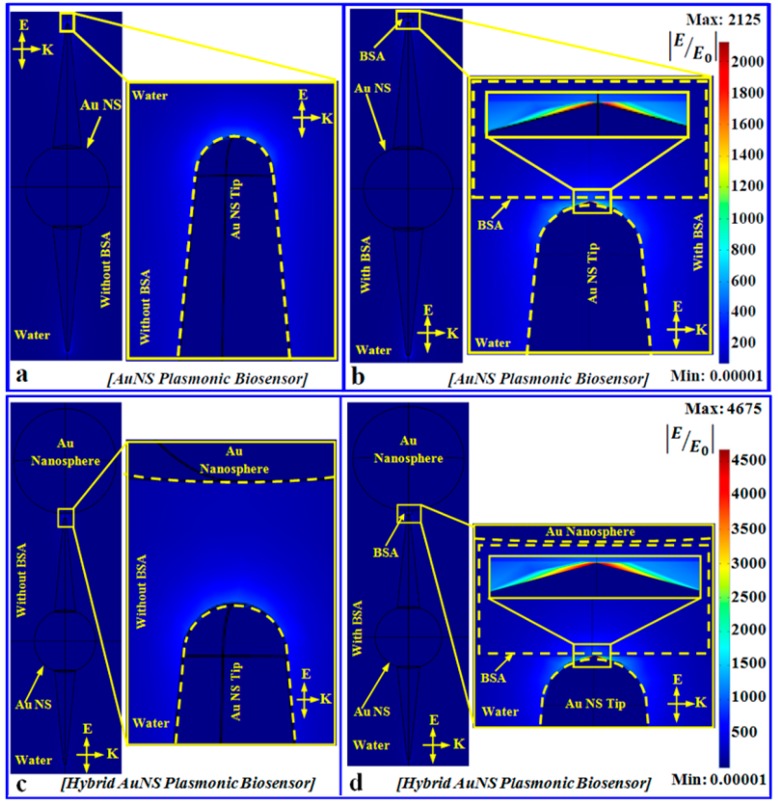
(**a**) Interaction of single AuNS plasmonic biosensor dispersed in water with incident light in the absence of a BSA molecule. The zoomed portion of the yellow box shows the intensity enhancement at the pinnacle of the spike. (**b**) Interaction of a single AuNS plasmonic biosensor with incident light in the presence of BSA in water media. (**c**) Photon interaction of a hybrid plasmonic biosensor developed from a single AuNS and an Au nanosphere in the absence of BSA immersed in water. (**d**) Interaction of a hybrid plasmonic sensor with incident light in the presence of BSA in water media. Here in all cases, the incident light wave has polarization along the z-axis and propagation along the +y direction. The AuNS has a spike length of 88 nm and a 60 nm core, and the size of the Au nanosphere is 100 nm.

Our synthesized AuNSs with sharp spikes can create great intensity enhancement at their hot spots after resonant light illumination. A hybrid plasmonic nanogap constructed by coupling Au nanosphere at the tip of the AuNS via the above-mentioned hot electron mediated method realizes an excellent nanoscale detector for single small protein molecules. This hybrid nanogap can be converted to a sensor by immobilizing a capture molecule (probe) at the nanogap to specifically recognize molecules at a point of care. Figure 5 shows the interaction of a single plasmonic nanogap with a BSA protein molecule. Panel (a) reports how a single plasmonic nanostar antenna behaves when it interacts with photons in water. Panel (b) shows how the situation is modified if a BSA molecule is located in close proximity to the tip. Panel (c) is similarly describing the photon interaction of a heterodimer composed of a nanostar with an 88 nm spike length and a 100 nm gold nanosphere separated by a distance of 3.4 nm in water. Panel (d) shows the same interaction in the presence of a single BSA protein molecule situated in the heterodimeric nanogap. For this investigation, the incident light polarization has been assumed to be along the z-direction, whereas the propagation direction has been assumed to be the +y-direction.

All the extinction properties corresponding to a single AuNS and an AuNS-nanosphere heterodimer plasmonic biosensor immersed in water in the presence and absence of a BSA molecule are shown in Figures S6 and S7, respectively. The sensitivity of the heterodimeric nanogap can be easily compared with the single AuNS tip sensitivity based on the FEM simulation results.

In the above FEM simulation results, the enhancement measurement is given in terms of the quantity $\left|E/E_{0}\right|$. However, the shift in the wavelength due to the adsorption of the protein molecule is proportional to the light intensity ($\frac{I}{I_{0}}={\left|\frac{E}{E_{0}}\right|}^{2}$) at the binding site. That is why it is better to interpret the results in terms of the light intensity enhancement. The intensity enhancement by a 33 nm AuNS spike (the smallest spike length) in water is approximately 1.6 × 10^3^, whereas the intensity enhancement for 88 nm spikes is approximately 2.5 × 10^5^ in water. We have evaluated that a single BSA protein molecule (molecular weight ~ 66.5 kDa) in water can produce a measurable shift (>1 nm) if it is located in close proximity of an 88 nm nanostar spike, having a sensitivity of about 1940 nm/RIU.

It is worth noticing that the remarkable tenfold increase of the light intensity enhancement in the absence of protein molecules within the heterodimeric gap with respect to a single nanostar spike. Strikingly, for a 3.4 nm heterodimeric gap (100 nm gold nanosphere-88 nm AuNS spike), the intensity enhancement was found to be 2.3 × 10^6^ in the absence of a BSA molecule in water. Hence, such a heterodimeric plasmonic sensor can detect a single BSA molecule with a sensitivity of about 5800 nm/RIU, and its sensitivity is expected to increase further by decreasing the size of the asymmetric tip-sphere nanogap.

## 4. Conclusions

To summarize, we reported the study of heterodimeric nanostructures for plasmonic sensing based on the formation of nanogaps between nanostar tips and nanospheres. The stable, highly tunable AuNSs were synthesized here via a simple, one-step, surfactant-free, wet-chemistry method with high yield and characterized via optical and EELS spectroscopic investigations. The formation of the nanogap is controlled and triggered by exploiting the light-induced hot-electron injection at the tip of the nanostars through the localized surface chemistry method. These hybrids and asymmetric plasmonic nanogaps can dramatically confine the incident electromagnetic field at their hot spots, irrespective of the incident light polarization. The numerical investigation supports the experimental results and predicted an ultrahigh sensitivity of this single molecule plasmonic biosensor (>5000 nm/RIU). This result will allow for the fabrication of nanogap sensors with high sensitivity and specificity for proteins and nucleic acids tests, which can find large use in point-of-care diagnostic technologies based on an easy and accurate optical readout.

## Supplementary Materials

Additional information including the UV−Vis−NIR spectroscopic results and STEM-EELS results of synthesized nanoparticles along with the FEM simulation results are available online at http://www.mdpi.com/2072-666X/9/12/664/s1.
