# Supplementary material for: Heterodimeric Plasmonic Nanogaps for Biosensing
Source: Micromachines (Basel). 2018 Dec 16;9(12):664. doi: 10.3390/mi9120664 (PMC6316515; doi:10.3390/mi9120664)
Supplement: Supplementary file 1 [file micromachines-09-00664-s001.pdf]

Supporting Information

Heterodimeric Plasmonic Nanogaps for Biosensing

Sharmistha Chatterjee [1,2,3], Loredana Ricciardi [2,3], Julia I. Deitz [4,5], Robert E. A. Williams [4], David W. McComb [4,5], and Giuseppe Strangi [1,2,3,*]

1 Department of Physics, Case Western Reserve University, 10600 Euclid Avenue, Cleveland, OH 44106, USA; chatterjee.bwd@gmail.com
2 CNR-NANOTEC Istituto di Nanotecnologia and Department of Physics, University of Calabria, 87036 Rende, Italy; loredana.ricciardi@unical.it
3 Fondazione con Il Cuore, via Roma 170, 88811 Ciro' Marina, Italy
4 Center for Electron Microscopy and Analysis, The Ohio State University, Columbus, OH 43212, USA; deitz.13@osu.edu (J.I.D.); williams.2156@osu.edu (R.E.A.W.); mccomb.29@osu.edu (D.W.M.)
5 Department of Material Science and Engineering, The Ohio State University, Columbus, OH 43210, USA
* Correspondence: gxs284@case.edu

A. Effect of Adding Silver Nitrate ($AgNO_3$) During Nanoparticle Synthesis:

The presence of Ag^+ ($AgNO_3$) is necessary in this synthesis process for the nanostar formation as it helps to expedite the anisotropic growth of Au branches on certain crystallographic facets [1]. Figure S1 describes the morphology of gold nanoparticles prepared without $AgNO_3$ (Figure S1 a) and with 100 μl $AgNO_3$ solution of 1 mM concentration (Figure S1 b). Here it can be seen that the synthesis process will give only polydispersed nanorods and nanospheres in absence of Ag^+ ($AgNO_3$). Upon increasing the amount of $AgNO_3$ during the synthesis will increase the length and number of spikes of gold nanostars (AuNS).

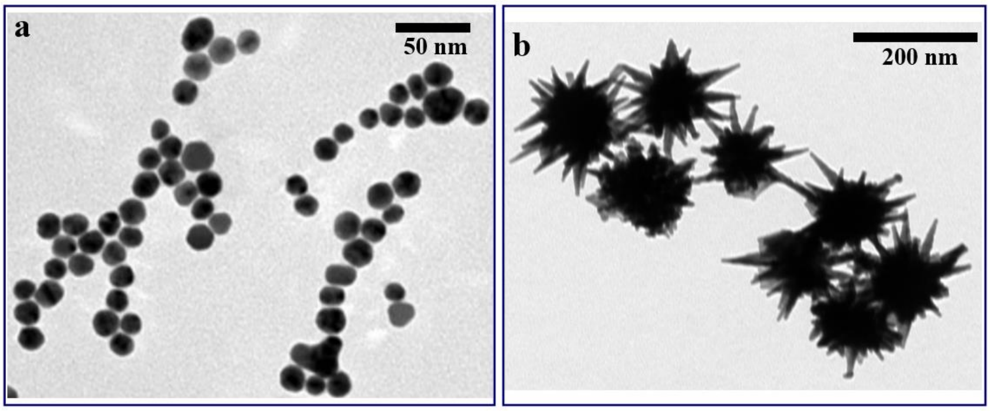

Figure S1. (a) A typical lower magnification randomly selected TEM image of a collection of Au nanoparticles produced without adding $AgNO_3$. (b) A typical lower magnification randomly selected TEM image of a collection of Au nanoparticles produced in presence of $AgNO_3$. Here we can see that synthesized gold nanoparticles in absence of $AgNO_3$ are mostly of spherical shape whereas in presence of $AgNO_3$ the nanoparticles turn out to be of star shape. This is showing that the presence of Ag^+ ($AgNO_3$) is necessary in this synthesis process for the nanostar formation.

B. Study on the Stirring Speed During the Synthesis Process:

A study carried out to probe the best stirring speed for the AuNS synthesis process reveals that 700 rpm is best for a successful AuNS synthesis as the measured extinction cross section for the AuNS solution is highest for that speed. The Figure below is showing the experimental extinction spectra for the AuNS solutions prepared using different stirring speed.

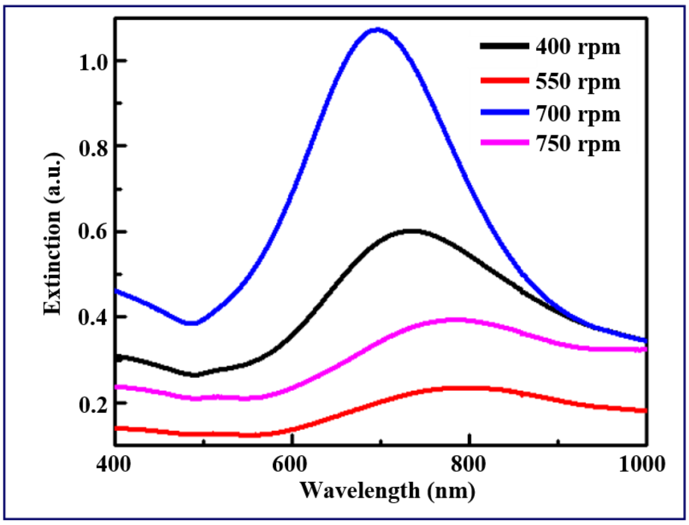

Figure S2. Comparison of different experimental UV-Vis-NIR spectra of the synthesized AuNS solutions produced using different stirring speeds. Here it can be seen that 700 rpm is the best stirring speed for a successful synthesis as the measured extinction cross section for that specific AuNS solution is highest.

C. Study on the Best Injection Time of Polyvinylpyrrolidone (PVP) During Synthesis:

To investigate the best injection time of PVP for a successful AuNS synthesis three AuNS solution has been prepared. The extinction spectra of all those three solutions are given in the Figure below. There the black curve is for that AUNS solution where PVP was added just before the addition of the ascorbic acid (AA) (before reduction). The red curve is for that AuNS solution where the PVP was added during the addition of AA (during reduction). The blue curve is for the AUNS solution where PVP was added after 2 minutes from the time of simultaneous injection of AA and $AgNO_3$ (after reduction). This AuNS solution (blue curve) has the highest extinction cross section and it is stable for more than 5 months in aqueous solution. Here, PVP is added to get a highly stable AuNS aqueous suspension by creating a hindrance to the nucleation process among the nanostars.

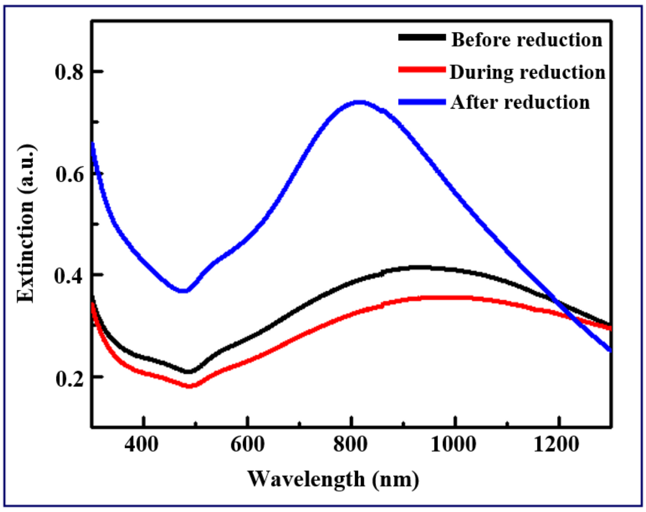

Figure S3. Comparison of different experimental UV-Vis-NIR spectra of the synthesized AuNS solutions is given where variation of PVP injection time has been taken into account. Here it can be seen that the best injection time of PVP for a successful synthesis is after 2 minutes from the time of simultaneous injection of AA and $AgNO_3$ not before or during the synthesis as the measured extinction cross section for that specific AuNS solution is highest.

D. Numerical Investigation on the Extinction Cross Section of Au Nanosphere of 60 nm size:

FEM simulation on the extinction property of the Au nanosphere of size 60 nm is shown in Figure below. Here it can be seen that the structure has localized surface plasmon resonance (LSPR) at 550 nm. During electron energy loss spectroscopy (EELS) study we have seen that the mode predominating in the area of the nanostars core was 2.2 eV mode which is equivalent to 564 nm. The size of the AuNS core is 60 nm. So the numerical study on the 60 nm Au nanosphere is supporting the fact that the 2.2 eV mode is originating at the core of the AuNS. During the FEM study, the surrounding media was taken as the water media, the incident light polarization was taken along z axis and propagation direction along +y axis. In this study the wavelength dependent permittivity of gold is taken from the Johnson and Christy measurements [2].

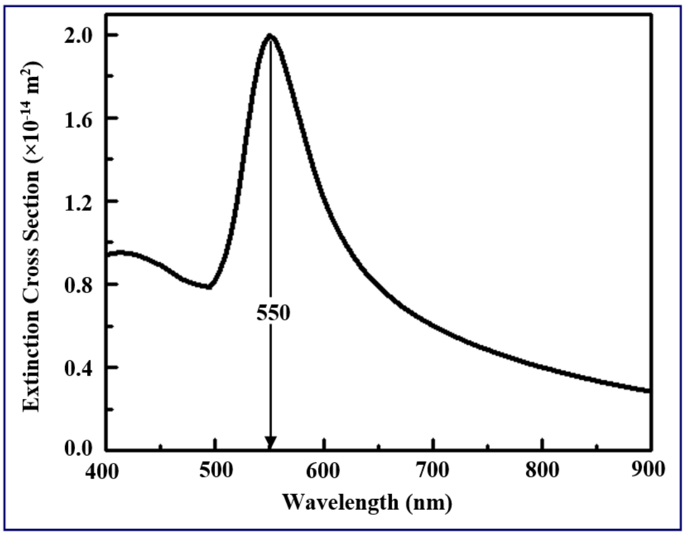

Figure S4. FEM analysis on light interaction of single Au nanosphere of size 60 nm immersed in water is shown here. The LSPR of 60 nm Au nanosphere is seen to occur at 550 nm.

E. STEM-EELS Investigations of the Synthesized Au Nanostructure:

Figure S5 (a) shows a STEM-HAADF image of the AuNS that was used for EELS analysis. The boxes on the image indicate the regions from which the EELS spectrum images were acquired. Figure S5 (b), S5 (c), and S5 (d) are showing the normalized EELS spectra of different regions of AuNS core and spike. The spectra from the centre of the nanostar were summed and the ZLP was removed using a reflected tail algorithm during EELS analysis. Here, the resulting low loss spectrum shows one major peak at 2.2 eV (~564 nm) in the region A which is in the core of the AuNS. Numerical investigation on Au nanosphere core of size 60 nm which has LSPR at 550 nm confirms that the 2.2 eV mode is originated at the core of AuNS.

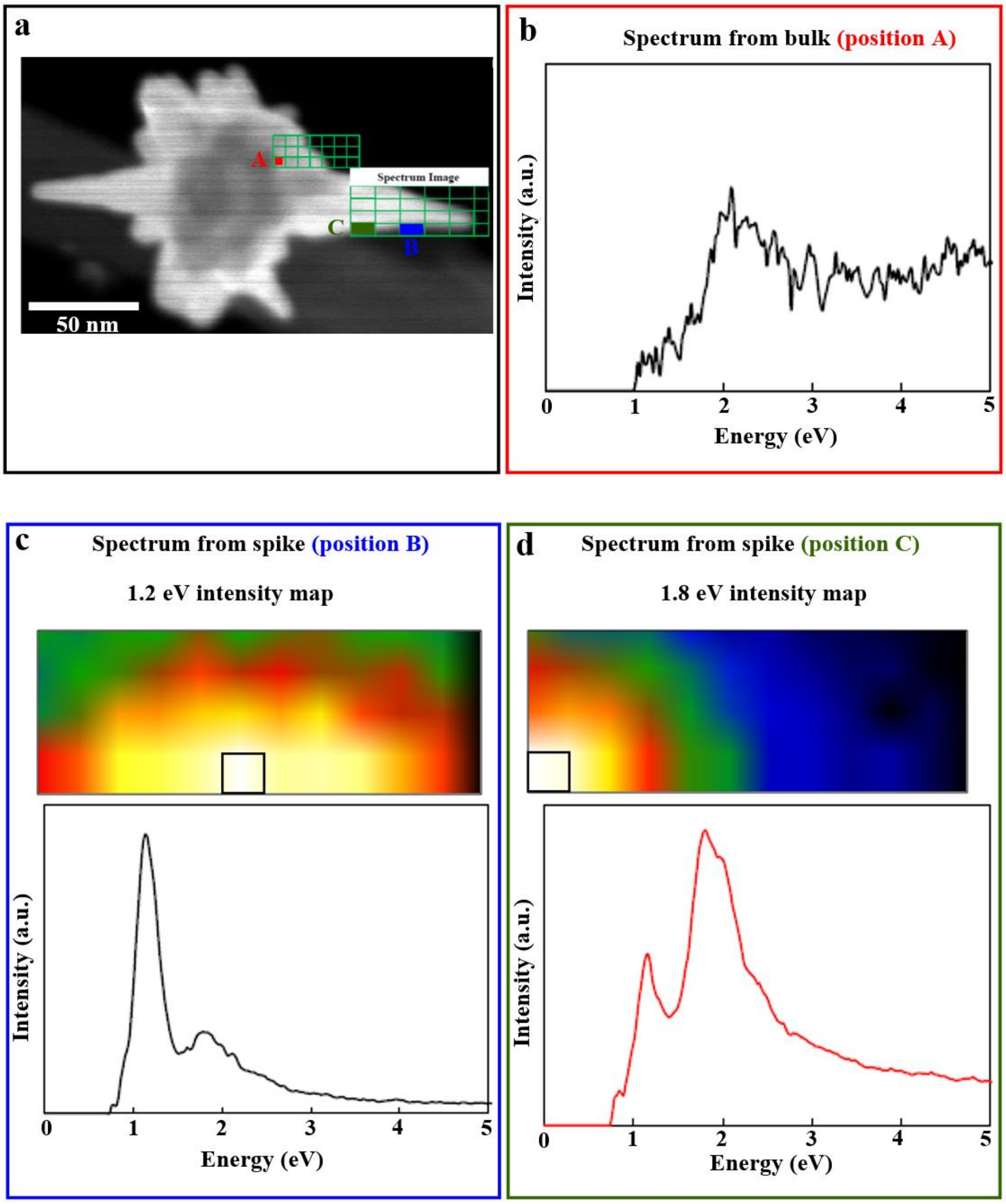

Figure S5. (a) Image of an AuNS taken for EELS analysis is shown. (b) EELS spectrum collected from position "A" which is in the core of the AuNS is shown. 2.2 eV mode is clearly appearing as the only mode from the core of the AuNS. (c) EELS spectrum collected from the position "B" which is approximately in the middle of the spike of the AuNS is shown. At this point the intensity of the 1.2 eV mode is highest with respect to its intensity at all the other regions of the spike. Here along with the 1.2 eV mode, the existence of 1.8 eV mode can also be observed. (d) EELS spectrum collected from the position "C" is shown. At this point the intensity of the 1.2 eV mode is lowest but the intensity of the 1.8 eV mode becomes highest with respect to their intensity at all the other regions of the spike.

The spectra from the spike, after ZLP subtraction, (Figure S5 c and S5 d) exhibit two major peaks at 1.2 eV (~1033 nm) and 1.8 eV (~689 nm). The relative intensity of the two peaks varies spatially along the spike. The peak at 1.2 eV shows a maximum intensity at a position approximately halfway along the length of the spike but the presence of 1.8 eV mode can also be seen there. On the other hand 1.8 eV peak exhibits maximum intensity at a location closer to the core of the nanostar but the presence of 1.2 eV mode can also be seen there. At the pinnacle of the 88 nm spike of AuNS the only mode that exists is the 1.2 eV mode (see Figure 3 in the manuscript). This information will be very helpful for hybridization of Au nanosphere to AuNS tip using the plasmonic hot electron driven non-localized surface chemistry method.

F. Numerical Investigation on the Extinction Property of Single AuNS with and without BSA:

FEM simulation on the extinction property of the AuNS of 60 nm core and 88 nm spike is shown in Figure below. Here the two situations have been considered – one is in absence of any protein molecule (Figure S6 a) and the other one is for the presence of a single Bovine Serum Albumin (BSA) molecule on the AUNS hot spot at its tip (Figure S6 b). According to the shown FEM analysis, the LSPR of a single AuNS plasmonic antenna immersed in water is found to occur at 1060 nm in absence BSA molecule. During EELS study the mode which predominated in the area of the AuNS tip was at 1.2 eV which is equivalent to 1033 nm. In presence of the single BSA molecule at AuNS tip the LSPR shifts to 1065 nm. So, according to the theoretical investigation, 5 nm wavelength shift is expected for adsorption of single BSA molecule (molecular weight ~66.5 kDa) for this AuNS.

During the FEM analysis, the incident light wave has polarization along z axis, propagation along +y direction and the orientation of the 88 nm spikes of the AuNS is taken along the axis of polarization. In this study the wavelength dependent permittivity of gold is taken from the Johnson and Christy measurements [2].

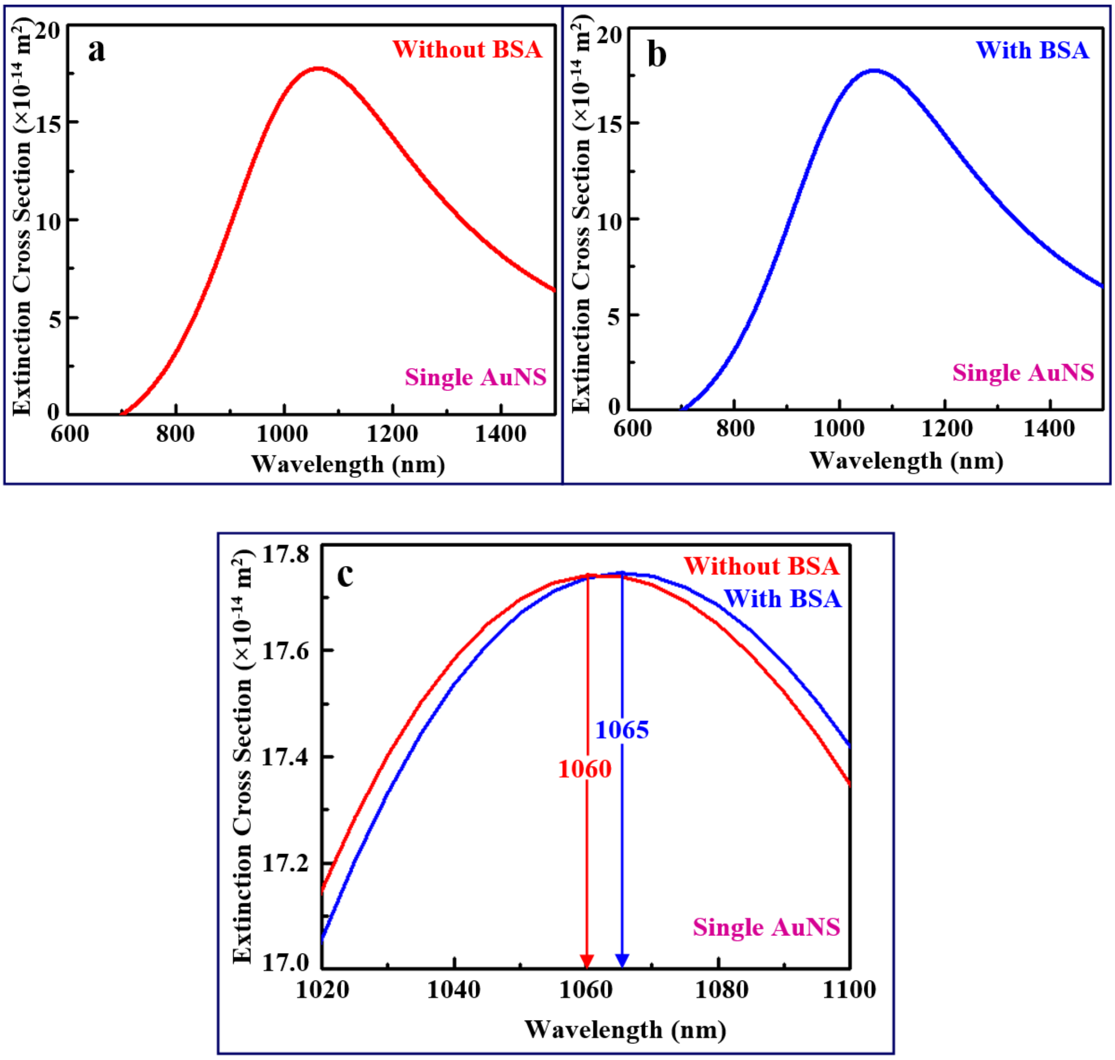

Figure S6. (a) Extinction property of single AuNS plasmonic biosensor dispersed in water in absence of BSA molecule is shown here. (b) Extinction property of single AuNS plasmonic biosensor in presence of BSA in water media. (c) Comparison between both the situation of single AuNS plasmonic biosensor, in absence and presence of BSA molecule. Here 5 nm shift is predicted according to the theoretical analysis for the adsorption of single BSA molecule at the AuNS tip.

G. Numerical Investigation on the Extinction Property of the AuNS-Au Nanosphere Heterodimer with and without BSA:

FEM simulation on the extinction property of the AuNS-Au nanosphere is shown in Figure below.

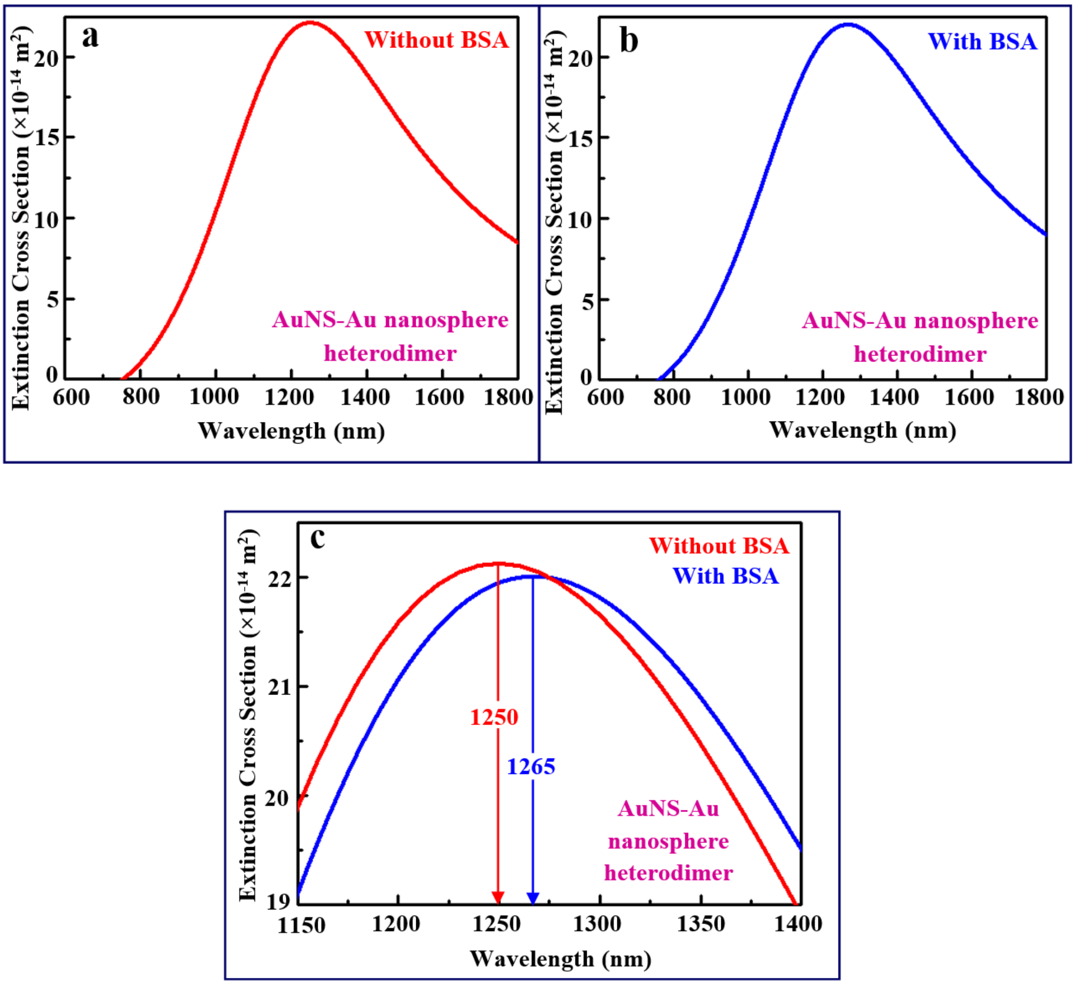

Figure S7. (a) Extinction property of AuNS-Au nanosphere hetero-dimer plasmonic biosensor dispersed in water media in absence of BSA molecule. (b) Extinction property of AuNS-Au nanosphere hetero-dimer plasmonic biosensor in presence of BSA. (c) Comparison between both the situation of heterodimeric plasmonic biosensor, in absence and presence of BSA molecule. Here, according to the theoretical analysis 15 nm shift is predicted for the adsorption of single BSA molecule at the hot spot created at the nanogap between AuNS tip and Au nanosphere.

Here the diameter of the AuNS core is 60 nm and the spike length is considered to be 88 nm. The diameter of the Au nanosphere is 100 nm. According to the shown FEM analysis, the LSPR of the AuNS-Au nanosphere hetero-dimer plasmonic antenna immersed in water is found to occur at 1250 nm in absence BSA molecule (Figure S7 a). The LSPR shifts to 1265 nm in presence of the single BSA molecule at the hot spot created at the nanogap between the AuNS tip and Au nanosphere (Figure S7 b). So, according to this theoretical analysis 15 nm wavelength shift is expected for adsorption of single BSA molecule (molecular weight ~66.5 kDa) for this AuNS-Au nanosphere hetero-dimer plasmonic biosensor.

During the FEM analysis, the incident light wave has polarization along z axis, propagation along +y direction and the orientation of the 88 nm spikes of the AuNS is taken along the axis of polarization. In this study the wavelength dependent permittivity of gold is taken from the Johnson and Christy measurements [2].

H. Numerical Investigation on the EELS intensity map of AuNS spike at 1.2 eV :

Figure **S8** below shows the numerical results on EELS intensity map of 88 nm AuNS spike based on finite element method (FEM). This plasmonic mode was experimentally measured via STEM-EELS experiment (see Figure 3 in the manuscript and **S5** in the supporting info) and it has been seen that for an 88 nm spike of AuNS 1.2 eV mode is the dominant mode in the tip region and could be useful for the local surface chemistry modification method. Although 1.2 eV mode is the dominant mode in the tip region of 88 nm spike yet the origin of that mode (the section of the nanostar spike where the intensity of 1.2 eV mode is highest) is not at the tip but little bit away from the tip. This is a one of the typical characteristic of a very useful breathing mode [3] which is not an ordinary plasmonic edge mode. This characteristic has also been verified by the numerical result which is shown in the Figure below. During numerical simulation we have checked the response of the nanostar for the incident wavelength equivalent to that incident electron energy. More simulation results and the experimental results used to probe this extremely useful breathing mode of the AuNS will be reported elsewhere.

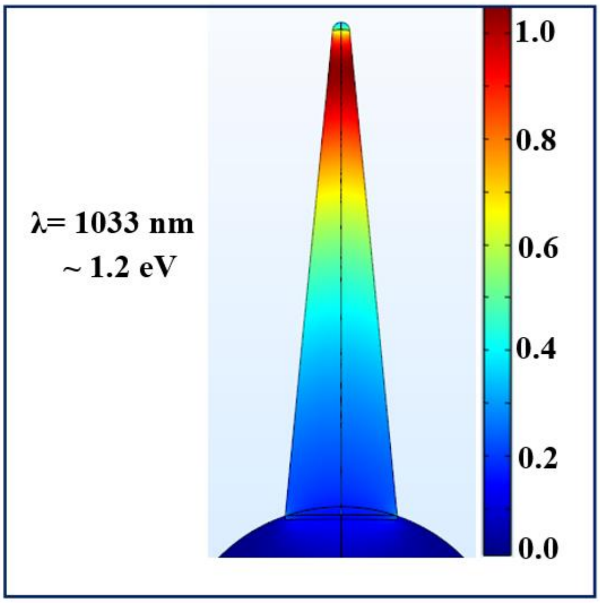

Figure S8: Finite element method simulations showing the response of the 88 nm nanospike at λ=1033 nm (~1.2 eV).

References

1. Yuan, H.; Khoury, C. G.; Hwang, H.; Wilson, C.M.; Grant, G. A.; Vo-Dinh, T. Gold Nanostars: Surfactant-Free Synthesis, 3D Modelling, and Two-Photon Photoluminescence Imaging. *Nanotechnology* 2012, *23,* 075102-075117.
2. Johnson, P. B.; Christy, R. W. Optical Constants of the Noble Metals. *Phys. Rev. B* 1972, *6,* 4370–4379.
3. Krug, M. K.; Reisecker, M.; Hohenau, A.; Ditlbacher, H.; Trugler, A.; Hohenester, U.; Krenn, J. R. Probing Plasmonic Breathing Modes Optically. *Appl. Phys. Lett.* 2014, *105,* 1711031–1711033.
